# Variation in Cooperative Behaviour within a Single City

**DOI:** 10.1371/journal.pone.0026922

**Published:** 2011-10-27

**Authors:** Daniel Nettle, Agathe Colléony, Maria Cockerill

**Affiliations:** 1 Centre for Behaviour and Evolution & Institute of Neuroscience, Newcastle University, Newcastle, United Kingdom; 2 Université de Rennes 1, Rennes, France; 3 School Improvement Service, North Tyneside Council, Wallsend, United Kingdom; University of Zaragoza, Spain

## Abstract

Human cooperative behaviour, as assayed by decisions in experimental economic dilemmas such as the Dictator Game, is variable across human populations. Within-population variation has been less well studied, especially within industrial societies. Moreover, little is known about the extent to which community-level variation in Dictator Game behaviour relates to community-level variation in real-world social behaviour. We chose two neighbourhoods of the city of Newcastle upon Tyne that were similar in most regards, but at opposite ends of the spectrum in terms of level of socioeconomic deprivation. We administered Dictator Games to randomly-selected residents, and also gathered a large number of more naturalistic measures of cooperativeness. There were dramatic differences in Dictator Game behaviour between the two neighbourhoods, with the mean allocation to the other player close to half the stake in the affluent neighbourhood, and close to one tenth of the stake in the deprived neighbourhood. Moreover, the deprived neighbourhood was also characterised by lower self-reported social capital, higher frequencies of crime and antisocial behaviour, a higher frequency of littering, and less willingness to take part in a survey or return a lost letter. On the other hand, there were no differences between the neighbourhoods in terms of the probability of helping a person who dropped an object, needed directions to a hospital, or needed to make change for a coin, and people on the streets were less likely to be alone in the deprived neighbourhood than the affluent one. We conclude that there can be dramatic local differences in cooperative behaviour within the same city, and that these need further theoretical explanation.

## Introduction

A striking finding of recent research on human cooperation is that its expression is highly variable. This has been shown most clearly by studies using experimental economic dilemmas, such as the Dictator Game (DG), across several societies [1], [2], [3], [4], [5]. In the DG, a participant has to divide a sum of money any way he wishes between himself and an anonymous stranger, thus providing a simple behavioural measure of generosity to others [6]. In the industrialised populations which have been studied, most participants allocate something to the other party, with a mean allocation between one third and one half of the stake [7]. In smaller-scale societies, though, the mean allocation is significantly lower [2], [5]. There have been rather fewer studies of within-population local variation, and those which have been carried out (e.g. [8], [9]) have mostly focussed on nonindustrial societies. Indeed, one can encounter the claim in the literature that there is not much within-population variation in pro-social behaviour to be found within industrialised nations ([8], p. 604). This conclusion may stem from reliance on relatively homogenous, affluent, university-related samples. Such reliance is typical of behavioural science more generally [10], [11]. However, contemporary Western cities may have neighbourhoods away from universities where cooperative behaviour is very different from how it is on campuses.

There are several reasons for believing this might be the case. Falk and Zehnder [12] used an experimental economic dilemma to show that participants from certain neighbourhoods of Zurich were trusted significantly less, and behaved in a significantly less trustworthy way, than participants from other neighbourhoods. Wilson et al. [13] used self-report measures of prosociality in a sample of young people from Binghamton, NY, and showed that the substantial variation in prosociality was spatially patterned, with detectable low- and high-prosociality areas. More generally, the survey-based sociological literature has revealed significant intra-population variation in social capital, which is usually defined as the social networks and norms that facilitate effective collective action [14], [15], [16]. Moreover, social psychologists have studied cooperation using field-experimental techniques, and observed significant heterogeneity, for example between different cities within the USA [17].

In this study, then, we sought to investigate the extent of neighbourhood differences in cooperative behaviour within one English city. England is a small but economically highly unequal country characterised by quite dramatic differences in vital prospects [18] and life-history parameters [19] between people of different socioeconomic positions. In cities, people are highly spatially assorted by socioeconomic position, and neighbourhoods can be classified on a continuum from deprived to affluent, using widely available indices. The literature would allow us to make predictions in either direction concerning differences in cooperation between affluent and deprived neighbourhoods. On the one hand, it is economically deprived communities who experience low perceived neighbourhood quality [13], high crime [15], [20], low social capital and trust [14], [15], [21], and low rates of civic participation [14]. These would suggest low levels of spontaneous cooperation in these areas. In the Zurich study, it was poor neighbourhoods which were characterised by low trust/trustworthiness in an experimental economic dilemma [12]. On the other hand, a recent US study showed that individuals of lower socioeconomic position were actually more generous in a DG and related measures of generosity than those of higher socioeconomic position [22]. The authors argued that people living under economic hardship are more dependent on one another for the achievement of their life goals, and hence develop greater concern for the outcomes of others, egalitarianism, and empathy (see also [23]). This literature would therefore suggest that we might find more willingness to cooperate with others in deprived than affluent neighbourhoods.

The main study reported here used two neighbourhoods about 6 kms apart, closely matched in key respects but differing sharply in the level of economic deprivation (see methods and Appendix S1 for background). The study had three goals. The first was to test whether there were significant differences between the two neighbourhoods in cooperative behaviour as assessed by the DG, and if so, in which direction, given the contrary predictions outlined above. The second goal was explore the robustness of the DG paradigm as a measure of cooperativeness. Repeated concerns have been expressed regarding whether the generosity observed in the DG stems from participants knowing that they are taking part in an experiment [24], [25], [26]. To mitigate this problem, we administered our DGs relatively surreptitiously, by inviting participants to take part in a social survey in their homes, for which they would receive £10 as a thank you. At the end of the survey, we asked them to indicate if they would like all of the £10 for themselves, or would prefer to have some it allocated to another party. This differs somewhat from the standard DG administration, but it recreates the essential DG dilemma without the participant necessarily being aware that their decision is itself a study variable. Another limitation of the DG is that the situation of having to share a resource with another person without knowing the identity of that person is presumably rare or nonexistent in real life. Gurven and Winking [27] have suggested that more insight into real-world cooperativeness might be gained with more ecologically realistic scenarios where the other party is not anonymous. Thus, as well as an anonymous DG (which we here term the ‘Unknown’ condition, since the identity of the recipient is unknown), we also used variants where the other party is a friend nominated by the participant (the ‘Friend’ condition), and where the beneficiary is a known good cause (the ‘Charity’ condition). If we still observe generosity in the DG despite the surreptitious administration, and if the patterns of allocation are similar whether the other party is unknown or is a friend, this will tend to support the robustness of the conventional DG as a behavioural measure of generosity.

The third goal of our main study was to validate the DG results against other measures of cooperation, including more naturalistic ones. Although we know that DG behaviour differs across human populations, we know relatively little about whether or how those differences are reflected in actual cooperation with others outside of the experimental situation. Studies which have tried to relate individual behaviour in experimental dilemmas to cooperativeness measured other ways have found correlations to be either absent or weak [27], [28], [29], [30]. The only study we are aware of which seeks to validate experimental dilemmas against more naturalistic measures of social cooperation at the community level is that of Lamba and Mace [9], who showed a weak positive correlation across villages between play in a public goods game, and social distribution of valued salt resources. To investigate the extent to which any neighbourhood DG differences mirror neighbourhood differences in cooperative behaviour more generally, we employed a range of other measures inspired by different traditions of research on social behaviour, such as those of sociology [14], [15], and social and environmental psychology [17], [31]. We used a *self-report survey* measuring social capital. Social capital has been the subject of extensive attention from sociologists, and is believed to be a key prerequisite for cooperative social action. The survey was administered to the same individuals as the DG, and if the DG is valid measure of cooperativeness, we might expect a positive relationship between DG allocations and social capital, at either the individual or neighbourhood level, or both. We also gathered *naturalistic observations* of cooperation-relevant behaviours in the neighbourhood: the number of crimes and antisocial behaviour incidents reported to the police over a four-month period, the frequency of dropping litter, the frequency of police patrols, and the mean group size of adults observed in the streets. Finally, we performed a series of *field experiments* to see if cooperation could be elicited more readily from strangers in one neighbourhood than in the other. The rate of response to our survey was one such measure. In addition, we measured the return rate of lost letters left on the pavement, the rate of spontaneous assistance when a researcher drops an object in the street, and the likelihood of help when a researcher asks a passerby to make change for a coin or give directions. One possibility is that all of the different measures will produce neighbourhood differences in the same direction as any difference seen in the DG. This would be a useful validation of DG methods as assays of cooperativeness at the community level, and also suggest that the many different traditions of research on cooperativeness (e.g. the social capital literature and the experimental economic dilemmas literature) are all measuring related underlying parameters. However, we are also open to the possibility that the different measures might produce different results. For example, field experiments similar to ours have previously been performed in 36 different US cities, with the finding that high cooperation on one measure, at the city level, does not predict high cooperation on all the others [17]. This suggests that cooperativeness, as a property of social groups, has multiple dissociable components. It is plausible, given the mutually contradictory predictions arising from previous literature, that our more deprived neighbourhood will be less cooperative than the affluent one on some measures and more on others.

The methods used in our main study are highly time-intensive, given that they involve behavioural observation and field experimentation as well as recruitment of experimental subjects. Thus, we have only been able to focus on two neighbourhoods, making it impossible to establish whether any differences we find are limited to our two study sites or part of a broader pattern of variation. To partially address this, in Appendix S2, we additionally present an ancillary study which used just a self-report survey, but recruited more broadly from 8 neighbourhoods within the same conurbation.

## Methods

Owing to the large number of methods used, we here provide only summary information, referring the reader to Appendix S1 for further details.

### Ethics statement

All components of this study were approved by the Faculty of Medical Sciences ethics committee at Newcastle University. Participants in the DG and self-report survey gave written consent to participate and for their anonymized data to be included in the analysis. For the naturalistic observations and field experiments, informed consent was not possible due to the nature of the study, and this requirement was waived by the ethics committee. However, all participants were in public spaces where they would have expected their behaviour to be visible to others, and no personally identifying information was recorded.

### Choice of study neighbourhoods

The two study neighbourhoods (A and B) have already been the site of ongoing behavioural research [32], [33]. They were carefully selected using the 2001 UK census and local piloting, to form a matched pair, similar in terms of physical layout, distance from the city centre, population size, density, and ethnic composition, but extremely divergent in terms of socioeconomic deprivation (see table 1). Neighbourhood A is in the 79^th^ percentile of all English neighbourhoods for socioeconomic deprivation (i.e. amongst the 22% most affluent), and neighbourhood B is in the 1^st^ percentile of deprivation (i.e. more deprived than over 99% of all English census areas). Individual-level characteristics of the residents, such as education and income, differ accordingly. For more information on the ethnographic background of the neighbourhoods, see Appendix S1.

**Table 1:** Key characteristics of the two study neighbourhoods.

	*Neighbourhood A*	*Neighbourhood B*
Total population (males)	3098 (1502)	3223 (1508)
Median age	37	34.5
Households	1250	1589
Population born in UK (%)	92	92
Index of Multiple Deprivation, score	8.74	76.43
Index of Multiple Deprivation, percentile of English neighbourhoods	79^th^	1^st^
Households owner-occupied	83%	18%
Residents in highest socioeconomic group of three-way classification (SEG-3)	74%	16%

Sources: 2001 UK census and 2004 Indices of Multiple Deprivation. IMD percentile is of all English census areas, where 1^st^ represents the most deprived 1%.

### Dictator Games

We randomly selected names and addresses of adults within the two neighbourhoods and posted a pack containing an explanatory letter and the self-report survey, along with a prepaid return envelope, through their doors (n = 170 in neighbourhood A, 230 in neighbourhood B). The survey itself is described below. The Dictator Game was surreptitiously administered on completion of the survey. The explanatory letter offered £10 in cash for survey completion, and an enclosed payment form asked the respondent to specify whether they would like the entire £10 to be delivered through their door, or would prefer to allocate any or all of it to another party (amounts in whole pounds only). Respondents were randomly assigned to one of three conditions. In the Unknown condition, the other party was described as a ‘randomly-chosen person from your local neighbourhood’. In the Friend condition, the participant was given a box in which to give the details of anyone they wished from the Tyneside area as the recipient, as long as that person lived at a different address. As we wished to make cooperation attractive in this condition, we doubled the monies allocated, so that by allocating all £10, the respondent could have their nominated friend receive £20. In the Charity condition, the recipient was specified as a locally well-known charitable foundation which provides free air ambulance services throughout the North of England. Again, allocations in this condition were doubled. All monies were delivered as promised, either in cash within one week with an accompanying letter to the person's house, or at the end of the study in the case of the charity donation.

### Self-report survey

The self-report survey participants filled in prior to the surreptitious DG contained basic demographic information, plus six questions probing social capital. These asked how much the respondent trusted people in the neighbourhood, how much they felt people in the neighbourhood looked out for one another, how well they knew their neighbours, and the extent to which they felt they have good friends locally (all answered on 7-point response scales). In addition, people were asked to list all those individuals they had contacted in the last two weeks for social reasons, and all those individuals they could turn to if there was a problem. We counted the number of individuals named (which was square-root transformed for analysis) in both cases.

### Naturalistic observations

*Crime and antisocial behaviour:* We also obtained data on all incidents of crime and antisocial behaviour reported to Northumbria police within each neighbourhood over the four months December 2010 to March 2011 from the police database at www.police.uk, classified by incident type. The remaining naturalistic measures were drawn from 12 hours of direct behavioural observation by a researcher on the streets of the each neighbourhoods, conducted between 19^th^ April and 8^th^ July 2010, one third on the main streets and the remaining two thirds in the residential streets (for details of sampling and recording, see Appendix S1). The total number of adults observed over the 12 hours was similar in the two neighbourhoods (Neighbourhood A: 4888, Neighbourhood B: 4750). We report the following measures. *Littering:* the number of times we saw a person drop bottles, cans, paper, cigarette ends or other trash onto the pavement. *Police patrols:* the number of times a police patrol, either on foot or in a motor vehicle, passed the researcher. *Social group size*: The number of adults in each social group observed. Groups were defined on the basis of moving or talking together.

### Field experiments

*Survey return rate:* We tracked the proportion of surveys returned, as this is itself a measure of willingness to cooperate with a request for help. Our other field experiments were derived from the previous social psychological literature on helping behaviour [17]. *Lost letter.* Following this well-established assay [26], [34], [35], a stamped sealed letter addressed to the first author at Newcastle University medical school was left on the pavement on rain-free mornings. Distances from a posting box were balanced across the two neighbourhoods. The proportion of letters arriving is a measure of strangers' willingness to do an act of anonymous kindness. Twenty-two letters were dropped in each neighbourhood. *Dropped object:* Research assistants (11 males, 13 females) walked in each neighbourhood and dropped a small personal item (e.g. keys, glove, pen) at 10 m from an oncoming lone pedestrian, seeming not to notice. Type and business of street, as well as sex and estimated age of target, were recorded. All research assistants completed the same number of trials in each neighbourhood. The target was classed as helping if he/she picked up the object or drew the research assistant's attention to it. Sixty objects were dropped in each neighbourhood in total. *Asking for directions:* The same research assistants approached a different target and asked for directions to a hospital which lay approximately 1 km from the study site. Targets were classified as helping if they gave detailed instructions on how to go to the hospital. There were 30 trials in each neighbourhood. *Making change:* The same research assistants approached a different target and asked for help to make change for a 50p or 20p coin. The target was classed as helping if they checked in their wallets or pockets. There were 30 trials in each neighbourhood.

### Analysis

In what follows, we report non-parametric statistical tests where assumptions of homogeneity of variance are violated, and parametric tests otherwise. We report rate ratios (RR) for frequencies of events in neighbourhood A versus B, and use Fisher's exact test (FET) to test for the significance of such differences. We use Fisher's combined probability test [36] to assess whether the naturalistic observations and field experiments as sets of tests of the null hypothesis of no neighbourhood difference allow us to reject that hypothesis.

## Results

### Dictator Games

**Figure 1:**
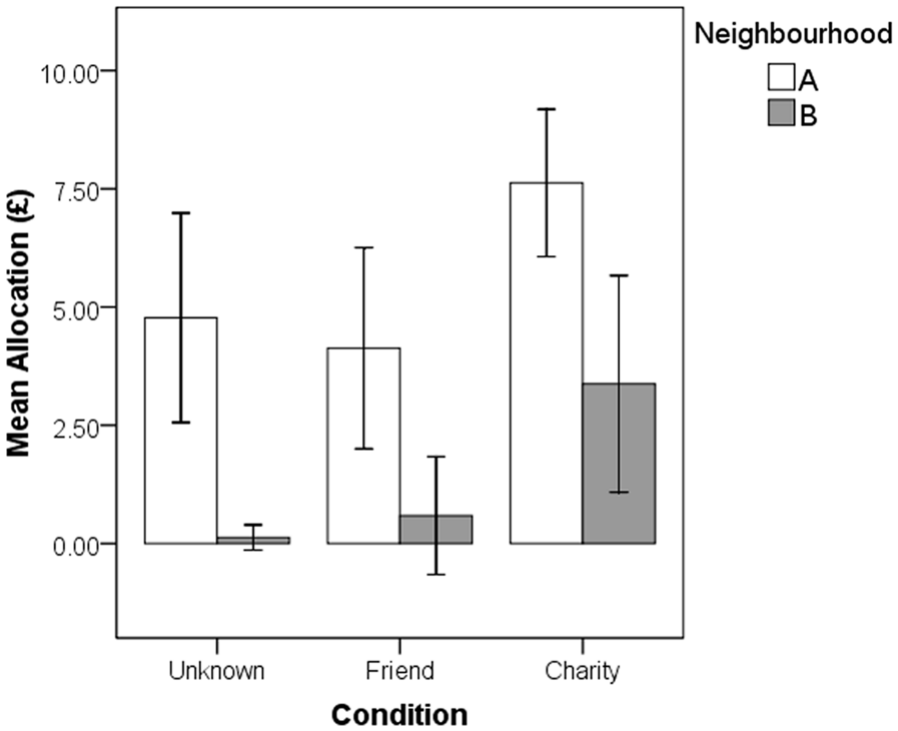
Mean Dictator Game offers for the two neighbourhoods across the three different conditions. Error bars represent 95% confidence intervals.

One hundred and eighteen people completed the DG (69 Neighbourhood A, 49 Neighbourhood B; 38 Unknown, 40 Friend, 40 Charity). The mean allocation to the other party was £3.81 (s.d. £4.64) of a possible £10. In a general linear model with condition and neighbourhood as predictors, there were significant effects of condition (F_2,112_ = 7.86, p<0.05, η^2^ = 0.12), and neighbourhood (F_1,112_ = 31.58, p<0.05, η^2^ = 0.22; see figure 1). The condition by neighbourhood interaction was not significant (F_2,112_ = 0.19, n.s.). The condition effect was due to generosity being significantly higher in Charity than in Unknown recipient (Tukey test, mean difference 3.11, p<0.05) or Friend (Tukey test, mean difference 3.30, p<0.05), though Unknown recipient and Friend did not differ from one another (Tukey test, mean difference 0.19, n.s.). The mean allocation across all conditions was £5.55 (s.d. £4.74) in neighbourhood A and £1.35 (s.d. £3.15) in neighbourhood B. Because the assumption of equality of variances was violated, we also conducted non-parametric Mann-Whitney U tests, which confirmed a significant difference between the two neighbourhoods overall, and in each condition separately (Overall: U = 927.5, z = −4.70, p<0.05; Unknown recipient: U = 93.5, z = −3.0, p<0.05; Friend: U = 122.5, z = −2.57, p<0.05; Charity: U = 94.5, z = −2.92, p<0.05).

### Self-report survey

One hundred and twenty-four people completed the self-report survey (74 Neighbourhood A, 50 Neighbourhood B). These comprised the 118 completing the DG plus 6 who did not return the payment form. The six social capital items were all significantly positively correlated with one another (*r*s 0.20–0.70), and all six showed a significant difference between the two neighbourhoods (see Appendix S1). Here, we standardized all six and summed them to produce an overall social capital index, which had high reliability (α = 0.81). This index differed strongly between the two neighbourhoods (Neighbourhood A: M 2.14, s.d. 3.11; Neighbourhood B: M −3.21, s.d. 3.82; t_121_ = −8.53, p<0.05; Cohen's *d* = 1.54).

Overall,there was a significant positive correlation between social capital and generosity in the DG (r_116_ = 0.40, p<0.05). However, this was driven by the differences between the neighbourhoods; the correlations between social capital and DG allocation within each neighbourhood were weaker and not significant (Neighbourhood A: r_68_ = 0.23, p = 0.06; Neighbourhood B: r_48_ = 0.13, n.s.). The lower social capital of neighbourhood B appeared to play a role in mediating the lower DG offers observed there; when social capital was added as a covariate to a General Linear Model predicting DG allocation from neighbourhood, both neighbourhood and social capital were significant predictors (Neighbourhood: F_1,113_ = 8.45, p<0.05; Social capital: F_1,113_ = 3.98, p<0.05), and the partial η^2^ for neighbourhood dropped from 0.20 to 0.07.

### Naturalistic observations

**Figure 2:**
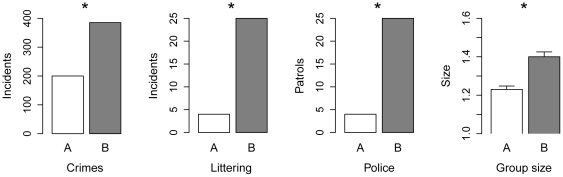
Summary of results from the four types of naturalistic observations across the two neighbourhoods. * significant difference in frequency using Fisher's exact test.

Figure 2 summarizes the naturalistic observations. *Crime and antisocial behaviour:* In neighbourhood A, there were 200 incidents reported to the police during the study period, compared to 385 in neighbourhood B (RR 0.52, FET against null hypothesis of equal crime rates, p = 0.0001). The magnitude of the difference varied with incident type, with violence and burglary showing the most markedly higher incidences in neighbourhood B (see Appendix S1). *Littering:* During the behavioural observation period, we observed 4 incidences of littering in neighbourhood A and 25 in neighbourhood B (RR 0.16, FET p = 0.005). *Police patrols:* We observed 4 police patrols in 12 hours in neighbourhood A against 23 in neighbourhood B (RR 0.17, FET p = 0.009). *Social group size:* We observed 3975 social groups containing adults in neighbourhood A and 3394 in neighbourhood B. Groups were significantly smaller in neighbourhood A than B (Means 1.23 vs. 1.40; Mann-Whitney U = 6014708.5, p<0.05). In Appendix S1, we show that this difference was reducible to a higher probability of adults being on their own in neighbourhood A compared to neighbourhood B, at all times of the day. This is despite the fact that the census tells us that the number of adults per household is actually higher in neighbourhood A than B (1.91 versus 1.52). As a set, the naturalistic observations allowed us to reject the null hypothesis of no difference between the neighbourhoods (Fisher's combined probability test, χ^2^ = 52.25, d.f. = 8, p<0.05).

### Field experiments

**Figure 3:**
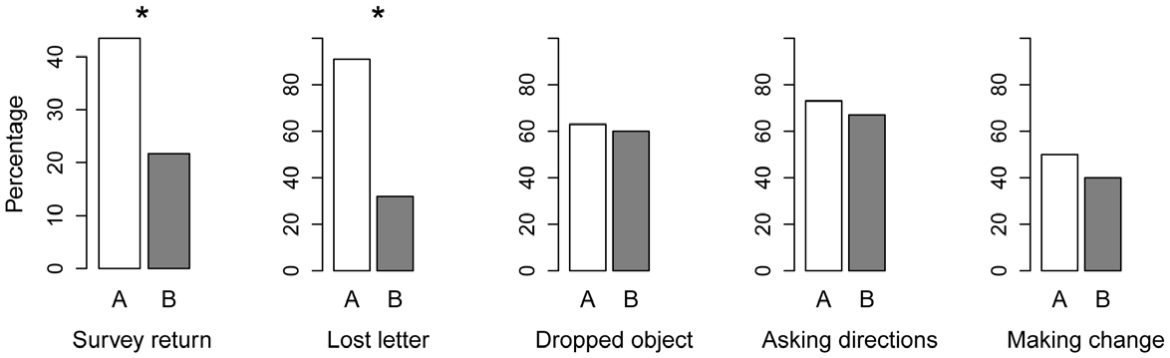
Summary of results from the five types of field experiment across the two neighbourhoods. * significant difference in frequency using Fisher's exact test.

Figure 3 summarizes the results of the field experiments. *Survey return rates:* Survey return rates were significantly different between the two neighbourhoods (43.5% v. 21.7%; RR = 2.00, FET p = 0.001). *Lost letter.* Of a possible 22 letters, 20 were returned from neighbourhood A and 7 from neighbourhood B (RR = 2.86; FET, p = 0.0001). *Dropped object.* Rates of helping were similar in the two neighbourhoods (A, 38/60, B 36/60; RR = 1.06; FET, p = 0.85). *Asking for directions.* 22 of 30 targets helped in neighbourhood A, compared to 20 in neighbourhood B (RR = 1.10, FET, p = 0.78). *Making change.* 15 of 30 targets helped in neighbourhood A, compared to 12 in neighbourhood B (RR = 1.25, FET, p = 0.60). As a set, the field experiments allowed us to reject the null hypothesis of no difference between the neighbourhoods (Fisher's combined probability test, χ^2^ = 34.08, d.f. = 10, p<0.05), albeit that this was driven entirely by the survey return rates and lost letters.

## Discussion

**Figure 4:**
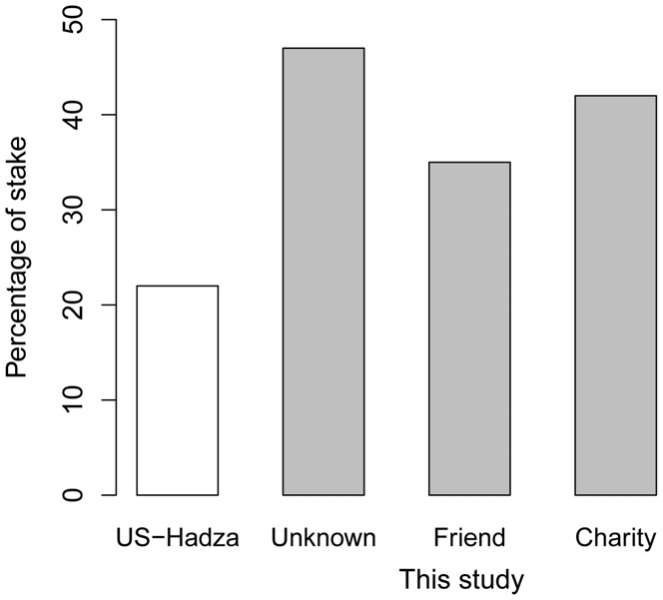
The difference, as a percentage of the available stake, between the mean DG allocation observed in a US sample and a sample of Hadza hunter-gatherers (white bar, from reference [**2**]), and between our neighbourhoods A and B for the three conditions of this study (grey bars).

The introduction outlined three objectives of our study. The first was to establish whether there was variation in DG behaviour across our two matched neighbourhoods within the same city. The results showed that there was, and that its magnitude was striking. In neighbourhood A, 60% of individuals gave something to the other party, whereas 6 kms away in neighbourhood B, only 20% of individuals did. This was even true in the Friend condition, where cooperation was made advantageous by doubling any money given, and the participant could choose the beneficiary. When the differences between the means observed for the two neighbourhoods are expressed as percentages of the available stake, they are substantially larger than the difference between a US sample and a sample of Hadza hunter-gatherers observed in a previous cross-cultural study (figure 4). With only two study neighbourhoods, it is impossible to ascertain how widespread discrepancies of this magnitude would be. However, in Appendix S2, we report an ancillary study where self-reported social trust was measured in almost 1,000 individuals in a further eight large Tyneside neighbourhoods. Social trust is one of our social capital variables, and, in the main study, correlates significantly with DG allocations (r = 0.39, p<0.05). In the ancillary study, we show that it varies substantially across the eight neighbourhoods, with 7% of the variation in trust at the between-neighbourhood level, and a continuum from high trust in the most affluent neighbourhoods to low trust in the most deprived. This suggests that the pattern of variation in DG offers we have observed in the main study might well generalize to the rest of the city.

In terms of the direction of the difference, it was the deprived neighbourhood B where DG offers were low. This accords with the findings of Falk and Zehnder in Zurich [12], but stands in contrast to the findings of Piff et al. [22], who found that individuals of lower socioeconomic position made more generous allocations in a DG than those of higher socioeconomic position. Our study population is different from that of Piff et al., and the deprived areas of Newcastle have a specific social history (see Appendix S1) which may not be shared by other places. There are also a number of key methodological differences between the studies. Piff et al.'s sample consisted of students at a major US university, and so it is unlikely that their sample contained the very wide range of socioeconomic positions our study reached. The relationship between socioeconomic circumstances and DG behaviour could well be non-linear. More importantly, in our study, individuals knew that recipients would be others from the deprived neighbourhood where they lived, whereas in the study of Piff et al., recipients were students drawn from the university community. These different set-ups are likely to produce different results; our study assorts individuals from a deprived area with one another, whilst theirs mixes individuals of different socioeconomic positions at random. Finally, in the Piff et al. study, DG decisions did not in fact have the exact financial consequences stated (i.e. there was deceit), which may have become known within the participant pool.

Our second objective was to explore the robustness of the DG methodology, given past concerns about the effects of the participant knowing that he or she is taking part in an experiment [24], [25], and the artificiality of dividing a resource with another party without knowing who that party is [27]. We used a relatively surreptitious administration, and, in the affluent neighbourhood A, found a rate of generosity which is in line with previous studies from affluent Western groups using a non-surreptitious administration, suggesting that awareness of taking part in an experiment is not prerequisite for generosity in the DG (cf. [26]). Moreover, changing the recipient to be a friend named by the participant had no significant effects on the level of generosity. Specifying the recipient as a charitable good cause did significantly increase giving. However, the relative difference between the two neighbourhoods was the same in all conditions, suggesting that as a community-level measure of cooperativeness, the DG is relatively robust to variation in how the dilemma is specified.

Most importantly, our study allowed us to validate the DG against other measures of cooperativeness at the community level. Neighbourhoods A and B differed markedly in DG offers, and they also differed in a whole suite of other ways (summarised in table 2) that can relate to the readiness of people there to cooperate with one another. The social capital of residents of neighbourhood A was much higher than that of neighbourhood B, and the social capital difference partially statistically mediated the difference in DG allocation between the two sites. Importantly for the validity of both the DG and the social capital survey as community-level measures, we found that in the neighbourhood where DG offers and social capital were relatively low, crime and antisocial behaviour were relatively frequent, especially violent crime and burglary, individuals were more likely to drop their litter on the street, and the police, no doubt aware of these differences, patrolled more heavily. Furthermore, in that neighbourhood, people were less likely to respond to a survey requesting their participation, and less likely to pick up a lost letter and see that it was mailed. Thus, these results all suggest that if one community is typified by lower DG offers and self-reported social capital than another, this does mean that everyday cooperation between members of that community is less widespread or forthcoming.

**Table 2:** Summary of the measures used and the results observed.

Measure type	Measure	Result
Experimental economic dilemma	DG	People in A give more than people in B, regardless of identity of recipient
Self-report	Social capital	All measures of social capital higher in A than B
Naturalistic observations	Crime and antisocial behaviour	Around half as many incidents reported to police in A compared to B; violence and burglary especially rarer
	Littering	People much less likely to drop litter in A compared to B
	Police patrols	Police patrol A at much lower frequency than B
	Social group size	Adults in the streets are *more* likely to be alone in A than adults in B
Field experiments	Survey return rates	People in A more likely to respond to a request to participate in a survey
	Lost letter	People in A more likely to mail in a stamped letter left on pavement
	Dropped object	No difference between A and B in likelihood of helping a stranger who has seemingly dropped something
	Asking for directions	No difference between A and B in likelihood of helping a stranger who needs to find the hospital
	Making change	No difference between A and B in helping a stranger who needs to make change for a coin

Some of our field experiments, namely dropped objects, asking for change and asking for directions, showed no significant difference between neighbourhoods. We had no prior expectation that this would be the case. We note that the experiments which show no difference involve face to face interaction, whereas survey return and lost letter do not. In effect, in the survey response and dropped letter, the participant can decide not to get involved in an interaction with a stranger at all, whereas in the asking for directions and asking for change, he or she is unavoidably involved by virtue of the fact that the experimenter has approached. Thus, it would make sense for there to be neighbourhood differences in the former set of tasks, but not necessarily the latter, if people in neighbourhood B are following a policy of avoiding initiating avoidable social interactions with people they do not know well. Previous studies using similar methods have also found that different types of helping do not all pattern together at the community level, and not all show a relationship with economic conditions [17]. Greater understanding the different types of helping, and how each relates to different aspects of the social context, is still needed (see [37]).

One measure showed a pattern somewhat contrary to the others, namely social group size. In the deprived neighbourhood B, where people named fewer others they could turn to in time of need, and where they were less likely to allocate any money to a friend in the DG, they were nonetheless more likely to be with someone else when moving around the neighbourhood. This was not because they were more likely to live with someone else, since there are in fact more lone-adult households in neighbourhood B than in A. One interpretation of this difference would be that in neighbourhood B, social ties are either strong (a few ties per individual), or completely absent, whereas in neighbourhood A, individuals have a greater range of weak ties which foster overall trust and community cohesion. This argument is in line with Granovetter's classic analysis of why working class-communities in Boston's West End failed to take collective action, despite containing strong individual friendships [38]. Admittedly, it would not explain the reluctance of residents in neighbourhood B to cooperate in the Friend condition of the DG, where only one friend needs to be named.

The interpretation of some of the differences listed in table 2 is debatable. For example, the £10 stake may be worth more to residents of neighbourhood B, where incomes are lower. However, previous research with the DG has found that the stake can be doubled [6], or even increased by an order of magnitude [39], with no significant effect on the pattern of allocation. Thus, it seems unlikely that the neighbourhood difference could be explained away by differences in the value of £10. It is also possible that the participants from neighbourhood B either understood the paradigm less well or trusted the researchers less to deliver the money than those of neighbourhood A. However, participants allocating everything to themselves had to indicate an active choice to do so, and it is unclear why lack of trust of the researchers – which would itself be a relevant finding, and is suggested by the lower survey return rate - should lead to a greater allocation to self rather than the other party. If you don't believe the researchers are likely to deliver, why not give all the money away, or simply not return the payment form?

Although the exact meaning of any individual measure may be equivocal so many different measures taken together do begin to reveal something of a pattern, and suggest that people's social experience and relations within these two nearby neighbourhoods are profoundly different. We feel these results are important both practically and theoretically. Practically, they confirm using a novel suite of methods that some socioeconomically deprived communities can fall into an equilibrium of low trust and low social investment. Such a situation affects people's quality of life, undermines civic and regeneration efforts [40], and allows disorder to flourish [15]. Experimental dilemmas such as the DG may have a role, as an alternative to traditional self-report surveys, as barometric measures for community-level social cohesion and connectedness, in attempts to understand and mitigate these dynamics.

Theoretically, our study adds to our growing understanding of the phenotypic variability in human cooperation. The results conform with those from recent studies of non-industrial populations showing that the within-population variation in cooperative behaviour can be just as marked and substantial as the between-population variation [9]. In particular, the claim that there is little within-country variation in prosocial behaviour in industrial societies ([8], p. 604) can clearly not be upheld, and may be an artefact of restricted participant pools. The results also suggest new avenues of investigation in terms of the causes of intra-community variation in cooperative behaviour. Existing approaches tend to invoke stable, societal-level culturally-transmitted norms [1]. However, our two study neighbourhoods are part of the same society, and their residents share the same broad cultural heritage. Yet, their DG behaviour is as divergent as any two groups yet studied. Thus, determinants operating at a more local level must be invoked. There are important differences between the neighbourhoods in terms of ecology and demography, and it may be that the differences in cooperative behaviour represent immediate evoked responses to these. In neighbourhood B, resources are scarce, and mortality and morbidity are high [19]. Many people are in poor health and material states, and their temporal discount rates and risk preferences will be likely to differ from those of their affluent neighbours. All of these factors should be expected to affect decisions about social investment. As for demographic processes, recent theoretical models have shown that the ability of individuals to leave locations where cooperation is low has a powerful influence on its stability [41], [42]. Neighbourhood A is inhabited by affluent owner-occupiers who have the resources to simply move away if local social behaviour is not to their taste. Neighbourhood B has experienced decades of selective outmigration by people with the means to do so, and its population has declined substantially (see Appendix S1). The remaining residents are largely those who have no means to exert any location choice. They thus have no real option but to find ways of accommodating to the locally prevailing patterns, which they may do by not initiating avoidable social encounters. This would entrench the pattern of low trust and small social networks which we observed.

## Supporting Information

Appendix S1 Additional background, methods and analyses.
(PDF)

Appendix S2 Ancillary self-report study.
(PDF)
