# Supplementary material for: Variation in Cooperative Behaviour within a Single City
Source: PLoS One. 2011 Oct 27;6(10):e26922. doi: 10.1371/journal.pone.0026922 (PMC3203179; doi:10.1371/journal.pone.0026922)
Supplement: Appendix S1 — Additional background, methods and analyses. (PDF) [file pone.0026922.s001.pdf]

Appendix S1: Additional background, methods and analyses

This appendix provides additional information, analyses and data to supplement the results reported in the main paper. Section 1 provides some ethnographic background to the study site. Section 2 gives additional information on how the study neighbourhoods were delineated and how the naturalistic observations and field experiments were carried out. Section 3 provides some additional analyses of the data from the main study, reporting more variables and covariates.

1. Ethnographic background

The city of Newcastle upon Tyne stands at the heart of a conurbation of around 900,000 inhabitants situated in the Northeast of England. Its growth was based on coal-mining, shipbuilding and other heavy industries, and port activity. These traditional sources of employment began to decline after the Second World War, a decline which became a collapse after the 1970s. Other types of economic activity, such as service industries, science and education have grown in their place, and parts of the city have become very affluent in recent years. However, this growth has been very spatially uneven, with the result that architecturally and historically very similar neighbourhoods in different parts of the city have taken very divergent trajectories in recent decades. A very large housing cost gulf has developed between the sought-after North of the city and the areas along the riverside. In particular, the area of the city containing neighbourhood B has been characterised by a long-term pattern of high unemployment, deprivation and physical dilapidation. This has been accompanied by demographic loss, with the population declining by around one third over the last few decades [1]. The area has developed a negative reputation locally, fuelling a vicious cycle of outmigration by residents with economic options, which increases the concentration of economic deprivation, and exacerbates the general sense of decline. In the words of one authority on the city's development:

> [The area's] reputation is legendary: ..... the 1991 riots; joyriders; drugs and violence; notorious criminal families. One small, telling example of this reputation that I have experienced is of officials going to a meeting [in the area] trying to avoid taking their own cars. Another example, illustrating the impacts of the image, is that people [in the area] say that employers and others discriminate against them by association, simply because of their address or postcode. The image has led to students being advised not to live there because it is considered unsafe, even dangerous. Discrimination and the area's reputation encourage those who can do so to leave. Indeed, it has been disconcerting for those running regeneration projects to find that if people are helped to get jobs they are inclined to move out. It doesn't help that the image seems to be starkly and graphically emphasised by the visual reality. Nowadays, there are parts [of the area] that look appalling, with boarded up and burnt out houses, cleared sites, barbed wire and shuttered shops. [1, p. 31].

The deprived parts of the city have been the site of many different urban regeneration initiatives funded by local and national government (non-exhaustive list, 1960s: Urban Aid; 1970s: Benwell Community Development Programme; 1980s: Tyneside Enterprise Zone; 1990s: West End City Challenge, North Benwell New Beginnings, Scotswood Regeneration, Reviving the Heart of the West End, New Deal for Newcastle West). The most recent initiatives in the part of the city containing neighbourhood B stem from the 1999 *Going for Growth* plan, and have involved large-scale demolition and rebuilding of tracts of housing, with a view to creating communities afresh, and attracting people not currently willing to live in the area. Although these successive initiatives have certainly improved many aspects of the environment and infrastructure [1], the demographic decline has not yet been decisively reversed, and the perception persists that social relations in the deprived areas of the city remain relatively

problematic. Previous studies have found that when neighbourhoods or villages have local reputations for low cooperation, these reputations tend to be borne out by experimental data [2,3], as indeed proved to be the case here. Our motivation for this and related studies within the Tyneside conurbation [4,5,6] was to understand the social dynamics of life under conditions of urban deprivation, and to provide data which we hope will be of use in community development and initiatives to improve wellbeing.

2. Additional methods information

2.1 Delineation of study neighbourhoods

The UK census divides all of England into small areas with populations of 1-2,000 people (known as Lower Super Output Areas, LSOAs). Using the census, we selected and defined two neighbourhoods around 4km from the city center of Newcastle upon Tyne, one to the North and one in the West End, each composed of two contiguous LSOAs (neighbourhood A= Newcastle upon Tyne 005C plus 006E; neighbourhood B= Newcastle upon Tyne 27D plus 29B). The data reported in table 1 of the main paper represent the combined census data from the two LSOAs which make up each study neighbourhood. The two neighbourhoods were chosen for long-term study because of their high degree of similarity by many measures. Both consist of a main shopping street, with supermarkets, smaller shops, cafés and bus routes to the city centre, backed on either side by streets of low-rise housing mainly built in the nineteenth and twentieth centuries. The population structure and ethnic composition of the two neighbourhoods are very similar, and the main (dramatic) difference is their contrasting economic fortunes of the two over the last sixty years or so (see above).

2.2 Methods for naturalistic observations

The survey return rate data were obtained simply by tracking how many surveys had been delivered versus returned. Crime and antisocial behaviour data were extracted from the police database at www.police.uk by downloading monthly incident statistics for each of the streets in our two defined study areas, and summing these. The remaining naturalistic observations (littering, police patrols, social group size) were obtained in the following way. We divided the time between 9am and 9pm into 24 half-hour segments, and on each available weekday over the period 19th April – 8th July 2010, DN visited each neighbourhood for a different time segment. Segments were completed in random order, but once a particular time of day had been sampled in one neighbourhood, it was sampled in the other neighbourhood as soon as possible (median delay 1 day; maximum 4 days). Neighbourhood A was the first to be observed for 14 of the segments, and neighbourhood B first for the other 10. All five weekdays were represented at least 3 times in the data for each neighbourhood. The researcher spent the first ten minutes of each segment walking the complete length of the main shopping street, and the remaining 20 minutes walking at normal speed along randomly varying routes through the residential streets. All data collection occurred on school days during the school term.

The researcher wore a digital voice recorder and made verbal notes for subsequent transcription. He noted for each social group encountered (i.e. passed within plain sight for long enough to be identified), the number of adults, children (i.e. under 16) and babies it consisted of. Groups were demarcated on the basis of members standing talking together, moving together, or involvement in a mutual game, and included if they were in the street, pavement, parks, plazas or courtyards open to the street. People inside buildings or vehicles were not recorded, and the same group was not re-recorded if encountered multiple times within a time segment. A number of behaviours not discussed here were recorded [see 5,6], plus (relevant to current purposes) all incidents of a person visibly letting fall litter

onto the street of pavement (littering), and all occasions when a police patrol (on foot or in a vehicle, including community support officers) passed within sight. The researcher also recorded all incidents of vandalism (defined as damaging, burning, or bombarding buildings or public structures), but the number of incidents seen (6 total, 1 in neighbourhood A and 5 in neighbourhood B) was too small for statistical analysis.

2.3 Methods for field experiments

Lost letters consisted of a single sheet of typed paper addressed to DN, contained in a stamped hand-written envelope addressed to DN at Newcastle University Medical School. AC dropped them on rainless mornings in one or the other neighbourhood, matching the distance from a posting box of the most recent drop in the two neighbourhoods. No more than two letters were dropped in a neighbourhood in one week, and never on successive days or the same exact location.

The remaining field experiments (dropped object, asking for change, asking for directions) were conducted by AC with a group of volunteer research assistants (total 11 male and 13 female researchers). Although research assistants completed the tasks variable numbers of times overall, every research assistant carried them out the same number of times in each neighbourhood. Times of day and day of week of experimental sessions were balanced across neighbourhoods. Objects dropped varied (but were usually balanced across neighbourhoods within research assistants) and included 38 gloves, 14 packets of tissues, 14 keys, and 13 pens. Within one experimental session, two objects were dropped, directions were asked for once, and help making change was requested once. A few minutes were allowed to pass between interventions, and different target individuals were selected for each one.

3. Additional analyses

3.1 Predictors of allocations in the DG

In the main paper, we show that there are significant effects of neighbourhood and condition on allocation to the other party in the DG. We also carried out general linear model with sex added as an extra factor, and age and time living in the neighbourhood as additional covariates. The overall model is significant ($F_{13,99}$=3.81, p<0.05, η^2=0.33). Table S1 shows that neighbourhood and condition remain the only significant predictors of allocation, with no effects of sex, age, or time living in the neighbourhood.

3.2 Inter-correlations and neighbourhood differences on the self-report survey

The self-report survey contained six measures of social capital, namely perception that others in the neighbourhood could be trusted (*trust*), how much they felt people in the neighbourhood looked out for one another (*looking out*), how well they knew their neighbours (*know neighbours*), and the extent to which they felt they have good friends locally (*friends locally*). In addition, people were asked to list all those individuals they had contacted in the last two weeks for social reasons, and all those individuals they could turn to if there was a problem. The variables derived from these last two items (*social contact*, and *social support*, respectively) were counts of the numbers of individuals named, and are square-root transformed for the purposes of statistical testing and derivation of the social capital index.

Variable	F-ratio	Partial η^2
Neighbourhood	$F_{1,99}$= 18.15*	0.16
Condition	$F_{2,99}$= 7.18*	0.13
Sex	$F_{1,99}$= 0.52	-
Age (covariate)	$F_{1,99}$= 0.54	-
Time living in neighbourhood (covariate)	$F_{1,99}$= 0.46	-
Neighbourhood * Condition	$F_{2,99}$= 0.20	-
Sex * Neighbourhood	$F_{1,99}$= 0.24	-
Sex * Condition	$F_{2,99}$= 0.73	-
Sex * Neighbourhood * Condition	$F_{2,99}$= 0.11	-

Table S1. Results of a general linear model with allocation in the DG as the outcome variable and neighbourhood, condition, sex, age, and time living in neighbourhood as the predictors. * p<0.05.

The inter-correlations between the social capital items are reported in table S2, which also shows the correlation of each with the derived social capital index. Table S3 gives the mean score for each item overall, and within each neighbourhood, also showing that there are substantial neighbourhood differences on all of the social capital items, with trust showing the most marked divergence.

	Looking out	Know neighbours	Friends locally	Social contact	Social support	Social capital index
Trust	0.70	0.52	0.46	0.30	0.30	0.77
Looking out		0.58	0.52	0.27	0.31	0.79
Know neighbours			0.45	0.20	0.21	0.69
Friends locally				0.42	0.37	0.75
Social contact					0.60	0.65
Social support						0.65

Table S2. Inter-correlations between social capital items, and between each item and the derived social capital index. All correlations are significant at $p<0.05$.

Variable	Overall	Neighbourhood A	Neighbourhood B	Neighbourhood Difference (*d*)
Trust	5.27 (1.65)	6.20 (0.85)	3.90 (1.59)	1.80
Looking out	4.60 (1.74)	5.35 (1.33)	3.50 (1.71)	1.21
Know neighbours	5.48 (1.76)	6.03 (1.30)	5.48 (1.76)	0.36
Friends locally	5.41 (1.84)	5.95 (1.42)	4.62 (2.11)	0.74
Social contact	11.65 (9.35)	14.20 (9.21)	7.80 (8.23)	0.73
Social support	6.23 (5.01)	7.32 (5.54)	4.57 (3.55)	0.59

Table S3. Means (standard deviations) overall and for each neighbourhood for the six social capital items. The difference between the two neighbourhoods for each measure is reported as Cohen's *d*. All neighbourhood differences are significant at $p<0.05$.

We ran a general linear model with the social capital index as the outcome variable, and neighbourhood, sex, age, and time living in neighbourhood as the predictors. There were significant effects of neighbourhood ($F_{1,114}=60.70$, $p<0.05$, $\eta^2= 0.35$) and age ($F_{1,114}=4.91$, $p<0.05$, $\eta^2= 0.04$), with social capital increasing with age (B=0.05). The effects of sex ($F_{1,114}=2.18$) and time living in neighbourhood ($F_{1,114}=0.06$), along with all interactions, were not significant.

3.3 Crime and antisocial behaviour

In the main paper, we show that there were more incidents of crime and antisocial behaviour reported in neighbourhood B than A during the study period. Here, we break down these incidents by incident type, showing the overall numbers and also the rate ratio for neighbourhood B versus A (table S4). Please note that the rate ratios in this table are reported the other way around from all others in this paper (i.e. a higher number means a greater excess in neighbourhood B compared to A).

**Incident type**	**Neighbourhood B**	**Neighbourhood A**	**Rate ratio**
All	385	200	1.93
Antisocial behaviour	186	108	1.72
Burglary	48	14	3.43
Robbery	2	0	-
Vehicle crime	15	20	0.75
Violent crime	34	6	5.67
Other crime	100	52	1.92

Table S4. Numbers of incidents occurring within the study neighbourhoods reported to the police, December 2010-March 2011. 'Antisocial behaviour' refers to miscellaneous incidents which disturb others and lead to a police callout, for example, public drunkenness, verbal harassment, vandalism, disturbing the peace, graffiti and public disputes.

3.4 Social group sizes on the streets

In the main paper, we show that social groups on the streets contain significantly fewer adults in neighbourhood A than B. Here, we dichotomised groups into lone adult versus multiple adult. The proportion of adult groups consisting of a lone adult is significantly higher in neighbourhood A than B (table S5). This is true overall, and also in each period of the day considered separately (table S5).

	Neighbourhood A	Neighbourhood B	Neighbourhood difference
Overall	3206/3975 (80.7%)	2388/3394 (70.4%)	χ^2 = 106.11
Morning (9-12am)	861/1018 (84.6%)	688/915 (75.2%)	χ^2 = 26.67
Afternoon (12-3pm)	943/1183 (79.7%)	729/1034 (70.5%)	χ^2 = 25.24
Late afternoon (3-6pm)	899/1069 (84.1%)	581/825 (70.4%)	χ^2 = 50.97
Evening (6-9pm)	503/705 (71.3%)	390/620 (62.9%)	χ^2 = 10.71

Table S5. Proportion (percentage) of social groups containing an adult in which he or she is the lone adult, for each neighbourhood, overall, and for each period of the day. All neighbourhood differences are significant at $p<0.05$.

3.5 Predictors of helping in the field experiments

The main paper shows that there were no significant neighbourhood differences for dropped object, asking for directions, or asking for change. We also ran additional logistic regression models including type of street (main or residential), how busy the street was (scale 1 to7), sex of target person, estimated age of target person, and sex of experimenter as additional predictors. For *dropped object*, the only significant predictor was whether the street was main or residential, with more helping in residential than main streets (exp(B)=4.69, $p_{wald}<0.05$). For *asking for directions*, there was a near-significant effect of age of target (exp(B)=1.05, $p_{wald}=0.06$), and all other predictors were nonsignificant. For *asking for change*, there were no significant predictors of helping.

References

1. Robinson F (2005) Regenerating the West End of Newcastle: What went wrong? Northern Economic Review 36: 15-42.
2. Gurven M, Zanolini A, Schniter E (2008) Culture sometimes matters: Intra-cultural variation in pro-social behavior among Tsimane Amerindians. Journal of Economic Behavior & Organization 67: 587-607.
3. Falk A, Zehnder C (2007) Discrimination and in-group favoritism in a citywide trust experiment. Working Paper, Institute for Empirical Research in Economics. Zurich.
4. Nettle D, Cockerill M (2010) Development of social variation in reproductive schedules: A study from an English urban area. PLoS ONE 5: e12690.
5. Nettle D (2011) Large differences in publicly visible health behaviours across two neighbourhoods of the same city. PLoS ONE 6: e21051.
6. Nettle D (2011) Behaviour of parents and children in two contrasting urban neighbourhoods: An observational study. Journal of Ethology in press.
