# Supplementary material for: Variation in Cooperative Behaviour within a Single City
Source: PLoS One. 2011 Oct 27;6(10):e26922. doi: 10.1371/journal.pone.0026922 (PMC3203179; doi:10.1371/journal.pone.0026922)
Supplement: Appendix S2 — Ancillary self-report study. (PDF) [file pone.0026922.s002.pdf]

Appendix S2: Ancillary self-report study

1. Introduction

Although our main study revealed large neighbourhood differences in a number of measures of cooperation, the fact that only two neighbourhoods were studied means we have no idea how general such differences are within the Tyneside conurbation. Moreover, even if there is substantial inter-neighbourhood variation, we cannot tell whether deprived neighbourhoods in general exhibit lower cooperation, since some difference between our neighbourhoods A and B other than the difference in their socioeconomic conditions could have been responsible for the results. The methods used in the main study are very time-intensive, and we have not yet been able to repeat them for a broader set of neighbourhoods. However, were able to gather self-report data from around one thousand young people (aged 9-15) from eight other Tyneside neighbourhoods, as part of a different project [see 1]. As an addendum to that questionnaire, we asked the social trust question ('How much do you think people in your neighbourhood can be trusted in general?'). In the main study, we have shown that responses to this question correlate highly with other self-report measures of social capital (see table S2). Moreover, in our main study, trust responses differed very strongly between neighbourhood A and B (table S3), and correlated significantly with DG allocations (r_{117}=0.39, p<0.05). Thus, if there are significant neighbourhood differences in trust responses across the eight neighbourhoods of the additional study, it suggests that there are likely to be more general differences in cooperative behaviour as well. By having eight neighbourhoods (of varying degrees of socioeconomic deprivation) in the additional study, we are able to test whether there is a general correlation between socioeconomic conditions and trust on Tyneside.

2. Methods

Participants came from eight schools from the eastern part of Tyneside, recruited for a separate study. The number and age-profile of participants varied from school to school in a manner which is random with respect to the objectives of this study (*n* per school 20-378). However, each school provided a cross-section of students of the particular ages they chose to work with, by having whole classes participate, and most schools provided several age groups. The total sample of 1149 students (596 female) was made up of 409 9-11 year olds, 396 12-13 year olds, and 346 14-15 year olds.

Students completed an anonymous online survey in their classrooms, during the school day, which included the trust measure analyzed here, alongside other items. We analyzed the response to one of these other items, life satisfaction ('How happy do you feel about your life overall?') as a comparison, below. All participants worked individually at a computer. The research was approved by the psychology ethics committee of Newcastle University, by the School Improvement Service of North Tyneside council, and by participating schools.

Due to the computerized administration of the survey, the response to the trust measure was given in a different way to the main study. The survey asked 'On a scale of 1 to 100, how much do you think people in your neighbourhood can be trusted in general?'. Responses were given by dragging a visual slider along a scale marked from 0 to 100, with 'Not at all' and 'Very much' anchoring the two ends. The initial slider

position was 0 and a numerical readout of the current slider position was given to one side. Life satisfaction was assessed on a similar 100-point scale.

The area of the conurbation which the respondent came from was established by a free text response. We matched this response to one of the 20 electoral wards of which the eastern borough of the conurbation is composed (2000 administrative boundaries). Electoral wards are larger geographical units than the neighbourhoods used for the main study (approximately twice the size). 104 students could not be assigned a ward because although they resided outside borough boundaries, or else their responses were insufficiently specific. In addition, there were 78 missing responses for trust and 53 for life satisfaction. For each ward, we obtained the Index of Multiple Deprivation (IMD) for 2000 [the most recent available data, 2]. Higher IMD scores indicate more economically deprived areas (observed IMD range 6.73-70.85).

The sample contained some respondents from 17 different wards, but several wards had very few cases (9 wards with fewer than 50). Thus, we amalgamated wards in such a way that no area had fewer than 50 respondents, using the principles (a) that wards were only amalgamated with adjacent wards; and (b) no wards whose IMD scores differed by more than 5 were amalgamated. This procedure produced a final set of 8 large neighbourhoods, each providing 65-233 respondents. The IMD for these composite neighbourhoods was calculated as the mean of the IMDs of the constituent electoral wards, weighted by the number of respondents that each constituent ward supplied.

We first performed a general linear model analysis, with sex and age group as fixed factors, and neighbourhood as a random factor. To test the effect of neighbourhood deprivation on trust, we used multilevel regression modelling with MLwiN [3], treating respondents as the level 1 units, and neighbourhoods as the level 2 units, and including age group and sex as level 1 predictors alongside neighbourhood IMD as a level 2 predictor. For the multilevel analysis, parameters were considered statistically significant based on the change in -2loglikelihood resulting from dropping them [4].

3. Results

The mean reported trust was 63.44 (s.d. 27.49). The general linear model revealed a near-significant effect of age group on trust ($F_{2,949}$ = 2.68, p=0.07, η^2 = 0.01), but no effect of sex ($F_{1,949}$ = 0.4, n.s.). However, there was a significant random effect of neighbourhood ($F_{7,949}$ = 2.68, p<0.05, η^2 = 0.07). Thus, neighbourhood accounted for around 7% of the total variation in trust (see figure S1, left panel). This stands in contrast to the situation for life satisfaction, where there were significant effects of age group ($F_{2,973}$ = 3.55, p<0.05, η^2 = 0.01) and sex ($F_{1,973}$ = 9.43, p<0.05, η^2 = 0.01), but not neighbourhood ($F_{7,973}$ =1.63, n.s.; see figure S1, right panel).

In the multilevel analysis, neighbourhood IMD was a significant predictor of individual-level trust (B=-0.47, s.e.(B)= 0.14, p<0.05). Note that the coefficient is negative, meaning that more deprived neighbourhoods have lower trust. There was some suggestion that the social gradient in trust may become stronger with age. Figure S2 shows neighbourhood mean trust (adjusted for sex) against neighbourhood IMD score, for the youngest and oldest age groups separately. There was trend for the slope of the regression line to be steeper in the 14-15 year olds than the 9-11 year olds (*z*=1.68, p<0.10).

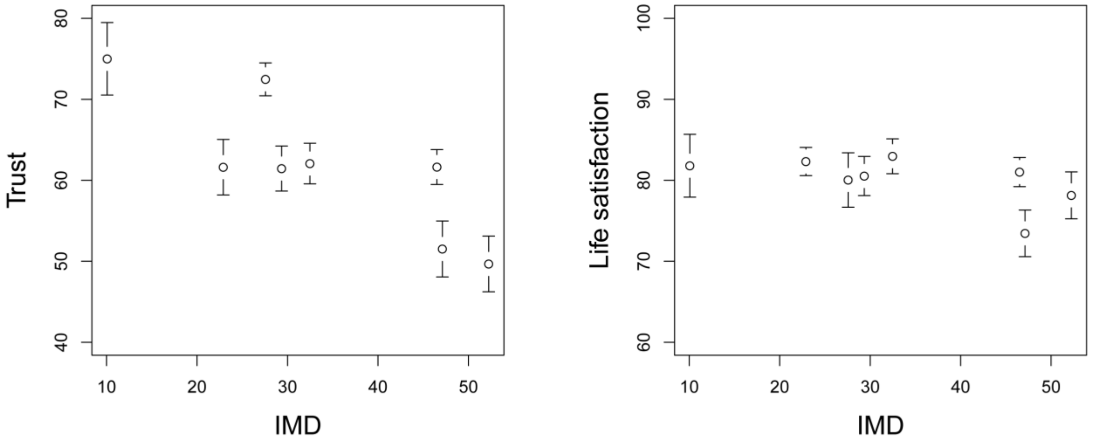

Figure S1. (left) Mean self-reported social trust for the eight study neighbourhoods, by neighbourhood index of multiple deprivation. (right) For comparison, mean life satisfaction for the same sample, against neighbourhood index of multiple deprivation. Error bars represent +/- one standard error.

4. Discussion

This additional self-report study revealed that there were substantial neighbourhood differences in young people's social trust across eight Tyneside neighbourhoods not included in the main study. Given that social trust was a good predictor of other social capital measures, and a significant predictor of DG allocation, in the main study, this suggests that neighbourhood differences in cooperative behaviour could be a general phenomenon in this conurbation. The neighbourhood differences are quite marked, with neighbourhood explaining around 7% of the variation in trust. Moreover, there appears to be something special about trust. Life satisfaction, another psychological variable which one would imagine reflects the local environment, does not show a similar pattern of between-neighbourhood variation (see figure S1). Presumably, social trust and other prerequisites for cooperative action are more heavily dependent on what others in the environs are doing than individual emotional state is.

Levels of trust across neighbourhoods are significantly though not perfectly predicted by the index of multiple deprivation in this additional study. This suggests that, as implied in the main paper, cooperative behaviour may be less readily sustained under conditions of economic deprivation.

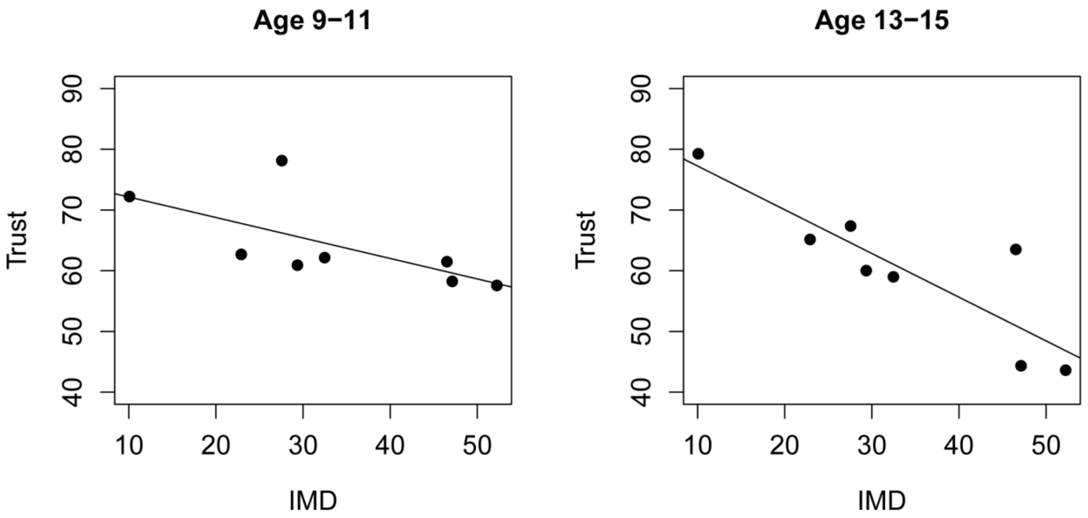

Figure S2. Relationship between mean social trust and neighbourhood index of multiple deprivation for 9-11 year olds (left), and 13-15 year olds (right).

References

1. Nettle D, Cockerill M (2010) Development of social variation in reproductive schedules: A study from an English urban area. PLoS ONE 5: e12690.
2. ONS (2010) Indices of Multiple Deprivation for Wards, 2000. London: Office for National Statistics.
3. Rasbash J, Charlton C, Browne WJ, Healy M, Cameron B (2010) MLwiN Version 2.19. Bristol: Centre for Multilevel Modelling.
4. Rasbash J, Steele F, Browne WJ, Goldstein H (2010) A User's Guide to MLwiN. Version 2.10. Bristol: Centre for Multilevel Modelling.
